# Deficits of Alzheimer’s Disease Neuropsychological Architecture Correlate with Specific Exosomal mRNA Expression: Evidence of a Continuum?

**DOI:** 10.3390/ijms26104897

**Published:** 2025-05-20

**Authors:** Ernesto Barceló, María I. Mosquera-Heredia, Oscar M. Vidal, Daniel A. Bolívar, Ricardo Allegri, Luis C. Morales, Carlos Silvera-Redondo, Mauricio Arcos-Burgos, Pilar Garavito-Galofre, Jorge I. Vélez

**Affiliations:** 1 Instituto Colombiano de Neuropedagogía, Barranquilla 080020, Colombia; erbarcelo@yahoo.com; 2 Department of Health Sciences, Universidad de La Costa, Barranquilla 080002, Colombia; 3 Grupo Internacional de Investigación Neuro-Conductual (GIINCO), Universidad de La Costa, Barranquilla 080002, Colombia; 4 Department of Medicine, Universidad del Norte, Barranquilla 081007, Colombiaoorjuela@uninorte.edu.co (O.M.V.); burbanoc@uninorte.edu.co (L.C.M.); mpgaravi@uninorte.edu.co (P.G.-G.); 5 Department of Industrial Engineering, Universidad del Norte, Barranquilla 081007, Colombia; 6 Institute for Neurological Research FLENI, Montañeses 2325, Buenos Aires C1428AQK, Argentina; rallegri@fleni.org.ar; 7 Grupo de Investigación en Psiquiatría (GIPSI), Departamento de Psiquiatría, Instituto de Investigaciones Médicas, Facultad de Medicina, Universidad de Antioquia, Medellin 050010, Colombia; mauricio.arcos@udea.edu.co

**Keywords:** Alzheimer’s disease, exosomes, mRNA, neuropsychological tests, biomarkers, predictive power score

## Abstract

Alzheimer’s disease (AD) is a neurodegenerative disorder characterized by cognitive decline and complex molecular changes. Extracellular vesicles (EVs), particularly exosomes, play a key role in intercellular communication and disease progression, transporting proteins, lipids, and nucleic acids. While altered exosomal mRNA profiles have emerged as potential biomarkers for AD, the relationship between mRNA expression and AD neuropsychological deficits remains unclear. Here, we investigated the correlation between exosomx10-derived mRNA signatures and neuropsychological performance in a cohort from Barranquilla, Colombia. Expression profiles of 16,585 mRNAs in 15 AD patients and 15 healthy controls were analysed using Generalized Linear Models (GLMs) and the Predictive Power Score (PPS). We identified significant correlations between specific mRNA signatures and key neuropsychological variables, including the Mini-Mental State Examination (MMSE), Montreal Cognitive Assessment (MoCA), Functional Assessment Screening Tool (FAST), Boston Naming Test, and Rey–Osterrieth Figure test. These mRNAs were in key AD-associated genes (i.e., *GABRB3* and *CADM1*), while other genes are novel (i.e., *SHROOM3*, *SLC7A2*, *GJB4*, and *XBP1*). PPS analyses further revealed predictive relationships between mRNA expression and neuropsychological variables, accounting for non-linear patterns and asymmetric associations. If replicated in more extensive and heterogeneous studies, these findings provide critical insights into the molecular basis governing the natural history of AD, potential personalized and non-invasive diagnosis, prognosis, follow-up, and potential targets for future therapies.

## 1. Introduction

Alzheimer’s disease (AD), a complex neurodegenerative disorder characterized by cognitive decline, memory loss, and the accumulation of amyloid plaques and neurofibrillary tangles in the brain [1], is the leading cause of dementia among older adults, with the number projected to reach 153 million people by 2050 [2].

While AD mechanisms are still being researched, extracellular vesicles (EVs), especially exosomes, are increasingly implicated in disease risk and progression [3,4,5]. These EVs are nano-sized membranx10-bound vesicles released by cells into the extracellular environment that mediate intercellular communication by transporting proteins, lipids, and nucleic acids [4,6]. EVs contribute to the spread of pathogenic proteins like amyloid-beta (Aβ) and tau, causing neuronal damage [4]. In addition, AD-derived EVs contain elevated levels of toxic proteins, and the EV composition is altered [6]. Thus, messenger RNAs (mRNAs), long non-coding RNAs (lncRNAs), and circular RNAs (circRNAs) present within EVs offer a rich source of information regarding AD pathobiology [3,4,7,8].

Research studies comparing the exosomal mRNA content between AD patients and healthy controls have identified potential biomarkers associated with disease progression and related conditions [9,10,11,12,13,14]. These studies often employ RNA sequencing techniques to analyze the mRNA profiles of EVs isolated from various biological fluids, including blood and cerebrospinal fluid [5,15], and hold promise for developing non-invasive diagnostic tests for AD [5]. Interestingly, differentially expressed mRNAs between individuals with AD and healthy controls are often associated with pathways implicated in AD pathogenesis, such as amyloidogenesis, tauopathy, neuroinflammation, and neuronal apoptosis [5,15]. More recently, our group identified several key mRNA transcripts associated with AD susceptibility and AD age of onset (ADAOO) [8].

Despite the promising findings from our and other research studies showing altered mRNA profiles in individuals with AD, and the potential of exosomx10-derived mRNA expression levels as non-invasive biomarkers for AD susceptibility and ADAOO prediction, the relationship between mRNA expression and the neuropsychological profiles of AD remains poorly understood. Although research in this area is still in its early stages, some studies suggest potential correlations. For instance, changes in exosomx10-derived mRNA levels associated with neuronal function and inflammation may be linked to deficits in memory, executive function, and other cognitive domains as assessed by neuropsychological tests [4,6].

Here, we hypothesize that specific exosomx10-derived mRNA signatures define the architecture of AD neuropsychological profiles outlined by language, memory, executive function, and praxis deficiencies. Using advanced data analytics tools, we study how the expression of 16,580 mRNA signatures correlates with AD neuropsychological domains and identify mRNAs that could serve as potential biomarkers of neuropsychological deficiencies in patients with AD and narrow down the potential ADAOO in those affected patients. While validation in more extensive and more diverse cohorts is crucial, our findings establish a framework to investigate how mRNA expression profiles correlate with distinct neuropsychological deficits in AD. This work bridges molecular findings with the natural history of the disease, personalized and non-invasive diagnosis, prognosis, and longitudinal monitoring strategies. Furthermore, these insights may accelerate the development of personalized therapies by prioritizing candidate targets for intervention.

## 2. Results

### 2.1. Subjects

We collected data from 30 individuals (22 [73.3%] females, 15 [50%] with AD) through our clinical evaluation protocols. Table 1 summarizes the results of the neuropsychological examinations. As expected, we identified statistically significant differences in key neuropsychological variables between healthy controls and individuals diagnosed with AD.

### 2.2. mRNA Signatures Contributing to Neuropsychological Manifestations of AD

**Figure 1:**
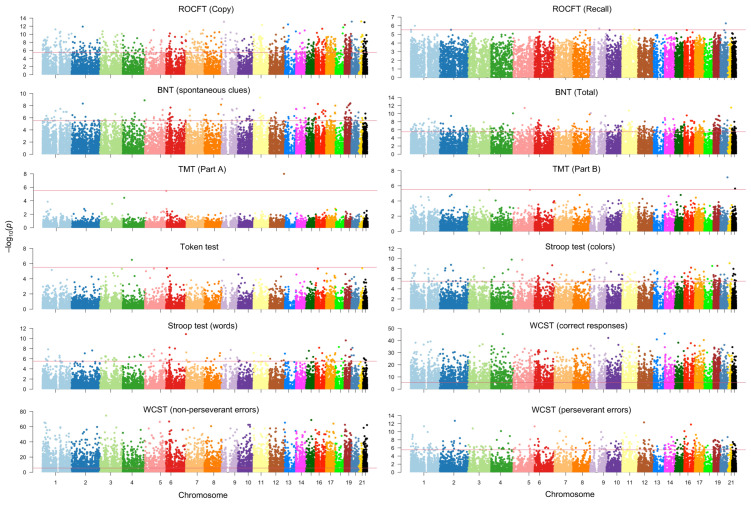
Manhattan plots showing mRNA signatures correlated with neuropsychological variables in a sample of individuals with AD and healthy controls from Barranquilla, Colombia. The horizontal red line corresponds to Bonferroni’s threshold. BNT: Boston Naming Test. Other conventions are in Table 1.

We quantified the expression of 16,585 mRNAs across all participants. A detailed analysis of these variables revealed that the expression of specific transcripts is associated with either enhanced or diminished performance. Figure 1 depicts the Manhattan plots for the neuropsychological variables with statistically significant results after correcting for multiple testing.

Table 2 reports the top mRNAs that are statistically significantly correlated with neuropsychological variables. We found 16 mRNAs to be statistically significantly correlated with the components of the ROCFT (Table 2). Some of these transcripts either increase or decrease the performance in the Copy or Recall components of ROCFT and are harbored in *TMEM239*, *XBP1*, *LCP1*, *SGTA*, *PDE2A*, *GJB4*, *PCSK5*, *DYNC2H1*, *TEKT4*, and *PRKCZ* genes (Table 2). For instance, higher expression levels of ENST00000361033 (*TMEM239*) are associated with a lower score in the Copy component of the ROCFT (Table 2). On the other hand, higher expression values of ENST00000295201 (*TEKT4*) increase the score in the Recall component of the ROCFT (Table 2).

A total of 157 mRNAs were potentially correlated with the Number of Spontaneous Clues. Regarding the Total Number of Correct responses, this number increased to 463 mRNAs (Table S1, Supplementary Material). Of these, mRNAs within the *RIN3*, *MMP2*, *PRTN3*, *PSMD5*, *CINP*, *CCDC70*, and *SLC7A2* genes are positively correlated with the Number of Spontaneous Clues of the Boston Naming Test, while expression in ENST00000004531 (*SLC7A2*) is associated with a decrease in the Total Number of Correct responses (Table 2).

Evaluation of the potential association between Parts A and B of the Trail Making Test (TMT) and mRNA expression identified three transcripts—MLEC, CATG00000053936.1 (*LAMA5*), and *PACSIN2*—that were associated with reduced performance in the TMT (Table 2). The expression of mRNAs located in the CATG00000114908.1 (*CDY2B*), *SHROOM3*, and *SAXO1* genes was found to be statistically significantly associated with performance in the Token test (Table 2). For instance, increased levels of MICT00000383608 (*CDY2B*) and ENST00000296043 (*SHROOM3*) are associated with poorer performance in the Token test, while increased expression of ENST00000380534 (*SAXO1*) correlated with better performance (Table 2).

Correlation analyses between mRNA expression levels and the Colors component of the Stroop test identified 157 statistically significant transcripts after correcting for multiple testing (Table S2, Supplementary Material). The most significant positive correlations with improved performance in the Colors test were observed for mRNAs associated with the *XBP1*, *MNT*, *MMP11*, and *CBX7* genes (Table 2). Conversely, mRNAs linked to the CATG00000066161.1 (*AMOTL2*), *SGTA*, *YKT6*, *IL12B*, *CATG00000036339.1* (*BCL2*), and CATG00000101329.1 (*EPPK1*) genes were negatively correlated (Table 2).

On the other hand, a total of 98 mRNAs were identified as significantly correlated with the number of words in the Stroop test after correction for multiple testing (Table S3, Supplementary Material). Table 2 shows the top 10 associated mRNAs. Specifically, mRNAs harbored in the *KEAP1*, *RPS16*, *ACO2*, and *MT4* genes are positively correlated with improved performance (Table 2). Conversely, mRNAs within the *EPS8L1*, *WISP1*, *C1QBP*, *CATG00000066161.1* (*AMOTL2*), *MEP1A*, and *GOSR2* genes were negatively correlated with performance (Table 2).

Finally, we identified several transcripts significantly correlated with an increased performance in the number of correct responses, non-perseverant errors, and perseverant errors of the Wisconsin Card Sorting Test (WCST) after correcting for multiple testing (Table S4, Supplementary Material). Table 2 reports the top 10 mRNAs. Although many transcripts are in genomic regions without annotated genes, these regions may still play significant roles in gene regulation and cellular function. Of particular interest are ENST00000055682 (*NEXMIF*) and ENST00000013807 (*ERCC1*), whose expressions are correlated with a lower number of perseverant errors (Table 2).

### 2.3. PPS of mRNA Signatures Across Neuropsychological Tests

**Figure 2:**
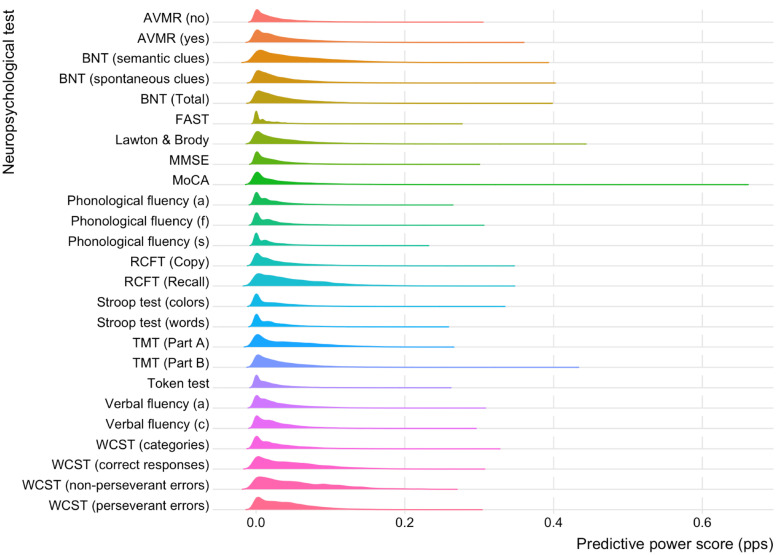
PPS distribution of mRNA signatures by neuropsychological test. BNT: Boston Naming Test. Other conventions are in Table 1.

Figure 2 shows the distribution of the PPS across all neuropsychological variables. As expected, these distributions are asymmetric. On average, 18.24% of mRNAs have a negligible PPS, implying that these transcripts offer no diagnostic power on the neuropsychological variables of interest. Among those with a PPS > 0, the minimum PPS value is 0.072 (BNT [semantic clues]) and the maximum is 0.361 (FAST).

Table 3 reports the top five mRNAs with the highest PPS for each neuropsychological variable. Some of these transcripts are harboured in genes associated with key biological processes generally disrupted in individuals with AD, and show decent predictive power for assessing the neuropsychological manifestations of AD. Across all neuropsychological variables, the mRNA with the maximum PPS across all neuropsychological was ENST00000311550 (*GABRB3*; PPS = 0.647) in MoCA, followed by ENST00000343289 (*NT5C2*; PPS = 0.439) in MoCA test, ENST00000299367 (*ATP6V1D*; PPS = 0.430) in Lawton and Brody, and ENST00000340116 (*ENOSF1*; PPS = 0.428) and ENST00000331581 (*CADM1*; PPS = 0.425) in MoCA (Table 3). Other identified transcripts with high PPS are harboured in genes to the pathophysiological changes typically observed in AD (i.e., *AMY2A*, *ANKH*, *ATP6V1D* and *B4GALT1*), genes associated to cognitive decline, memory impairment, and other neuropsychological manifestations in AD (i.e., *MECP2*, *S100B*, *GABRB3*, *BTBD16* and *AP003108.2*), and neuroinflammation (i.e., *S100B*, *CTLA4* and *CARD6*) (Table 3).

## 3. Discussion

In this study, we investigated the relationship between exosomes-derived mRNA signatures and the neuropsychological manifestations of AD in individuals from Barranquilla, Colombia. Comparison between individuals diagnosed with AD and healthy controls revealed important differences in cognitive performance as measured by several neuropsychological tests, including the Mini-Mental State Examination (MMSE), Montreal Cognitive Assessment (MoCA), Functional Assessment Screening Tool (FAST), Boston Naming Test (BNT), Verbal Fluency, Phonological Fluency, Trail Making Test (TMT), Rey–Osterrieth Complex Figure (ROCFT), Stroop test and one of the components of the Wisconsin Card Sorting test (WCST)(Table 1).

Analysis of mRNA transcripts using Generalized Linear Models (GLMs) identified significant correlations between mRNA expression levels and neuropsychological test performance in this cohort (Figure 1; Table 2). Several of these mRNAs are typically altered in AD, extending prior research on exosomal mRNA as potential biomarkers for AD [3,8,16,17,18,19]. Our findings suggest that changes in exosomal mRNA expression may contribute to the cognitive deficits characteristic of AD [9,20,21,22]. While some of these mRNAs are encoded by genes previously linked to AD-related processes, others are novel (Table 2 and Figure 1).

*SLC7A2* plays a role in arginine metabolism, and its dysregulation is linked to AD through neuroinflammation and oxidative stress [23]. Arginine transport is important for nitric oxide synthesis, which affects vascular function and neuroinflammatory pathways. Reduced SLC7A2 expression may worsen inflammation and neuronal damage, leading to cognitive decline in AD.

*PDE2A* is crucial for regulating cAMP and cGMP homeostasis and is highly expressed in brain regions critical for socio-cognitive behavior that are vulnerable to AD [24,25]. Overexpression of PDE2A impairs mitochondrial function and causes extensive mitochondrial fragmentation in neurons, which can be an early indicator of AD [25]. *PDE2A* inhibitors, especially those targeting mitochondrial PDE2A2, are under NIH-funded investigation as potential treatments to mitigate memory loss and nerve damage in AD [25].

*SGTA* has emerged as a protein of interest in AD due to its multifunctional role in cellular processes potentially relevant to neurodegeneration [26,27]. SGTA, a co-chaperone protein, is implicated in AD due to its roles in apoptosis, synaptic transmission, protein homeostasis, and amyloid processing, which is central to AD pathology and progression [26,28].

*SHROOM3* regulate axxonal guidance and cytoskeletal organization, which are critical for maintaining neuronal integrity in AD [29]; *GJB4* encodes connexion proteins involved in gap junctions; its altered expression disrupts neuronal communication [29]; *PCSK5* influences amyloid precursor protein (APP) processing, thereby affecting Aβ aggregation [30]; *DYNC2H1*, a dynein motor protein gene, is linked to intracellular transport and tau pathology [31]; *TEKT4*, associated with cytoskeletal organization, may influence synaptic stability [29]; and *PRKCZ* modulates synaptic plasticity and memory, correlating with cognitive decline in AD [29,31].

*RIN3* impacts APP trafficking and Aβ clearance, while *MMP2* and *MMP11* promote extracellular matrix remodelling and neuroinflammation and may exacerbate neuronal damage [30]. *KEAP1*, on the other hand, regulates oxidative stress via NRF2 signalling, contributing to neuronal vulnerability [32]. While *IL12B* drives neuroinflammation through microglial activation [30], *XBP1*, a key regulator of the unfolded protein response (UPR), worsens endoplasmic reticulum stress and neuronal death in AD [30,32]. Furthermore, mitochondrial dysfunction is affected by *ACO2*, which impacts energy metabolism critical for neuronal survival [32]. Finally, *C1QBP* influences immune responses and synapse pruning, further contributing to neuroinflammation in AD [30]. Notably, our findings highlight the multifaceted genetic mechanisms underlying AD pathology, emphasizing the relevance of mRNA expression in these genes to shaping cognitive performance in individuals with the disease. Validating these associations experimentally and exploring their therapeutic potential remains critical for advancing our understanding of AD.

We used the Predictive Power Score (PPS) to evaluate the predictive relationships between mRNA expression and neuropsychological variables. Unlike traditional correlation analyses, PPS accounts for non-linear patterns and asymmetric associations [33,34]. This analysis identified mRNAs associated with cognitive performance in AD (Table 3 and Figure 2). Key transcripts are harboured in *NTM2*, *GABRB3*, *HK1*, *TRIM7*, *SCAMP5*, *FOXF1*, *NT5C2*, and *CADM1*, which are involved in mechanisms underlying AD pathology.

ENST00000378165 (*NMT2*) was associated with the FAST screening tool (Table 3). *NMT2* encodes an enzyme crucial for cellular signalling and protein stability. NMT2 dysregulation may disrupt neuronal function and worsen proteostasis, impairing cognition, accelerating AD progression, and impairing memory and cognition. Protein modification pathways are increasingly implicated in neurodegenerative diseases, highlighting their potential role in AD pathogenesis [35,36,37].

*GABRB3* is essential for inhibitory neurotransmission. We previously reported that the ENST00000311550 (*GABRB3*) was a key predictor of AD diagnosis [8]. Here, this mRNA contributes to performance in FAST, MoCA, and Verbal Fluency (Table 3). Altered *GABRB3* expression may impair synaptic function, contributing to cognitive deficits in AD. Dysregulated GABAergic signalling has been associated with memory impairment and executive dysfunction, further implicating its role in AD pathology [29,37].

*HK1*, regulating glucose metabolism for neuronal energy, is crucial since impaired glucose metabolism is a feature of AD; HK1 dysregulation intensifies bioenergetic deficits and contributes to cognitive decline [35,38]. The finding that ENST00000643399 (*HK1*) predicts MoCA (Table 3) is critical for understanding cognitive impairment and early dementia signs in our population.

We identified that ENST00000274773 (*TRIM7*) has a significant predictive power of several neuropsychological tests (Table 3). *TRIM7* is involved in protein degradation and immune responses. Thus, its dysregulation could amplify neuroinflammation and impair protein clearance pathways central to AD pathology. The role of *TRIM7* in proteostasis highlights its potential as a therapeutic target [35,39].

*SCAMP5* regulates vesicular trafficking critical for synaptic function. Altered expression impacts APP processing and Aβ production [29,37]. *FOXF1*, on the other hand, influences cellular differentiation and survival, and its dysregulation may impair neuronal development and intensify neurodegeneration observed in AD brains. The fact that mRNAs within this gene have relevant predictive power in BNT and MMSE (Table 3) highlights its role in the neuropsychological manifestations of AD.

ENST00000343289 (*NT5C2*) is an essential predictor of the MoCA test (Table 3). *NT5C2* encodes a cytosolic 5’-nucleotidase involved in nucleotide metabolism. Impaired function could disrupt neuronal homeostasis and exacerbate oxidative stress in AD neurons [29,35], which may explain its association with this screening test in our sample. In addition, we identified ENST00000278483 (*HIKESHI*) may predict the results of both the Token and Stroop tests (Table 3). *HIKESHI* facilitates nuclear transport of heat shock proteins under stress conditions. Its dysregulation may impair proteostasis and protein aggregation, contributing to cognitive decline [38,39].

ENST00000300093 (*PLK1*) and ENST00000540200 (*POLDIP2*) were significant predictors of the Stroop test (Table 3). *PLK1* regulates cell cycle progression and DNA damage repair. Altered expression may contribute to neuronal apoptosis observed in AD brains, impacting executive function [35,40]. *POLDIP2* is involved in DNA replication and repair, such that impaired function increases genomic instability and intensify neurodegeneration observed in AD neurons, thus affecting executive function [35,40].

ENST00000375259 (*SLC35D2*) was identified as an essential predictor of Verbal Fluency (Table 3). *SLC35D2* is involved in glycosylation processes critical for protein folding and stability. Thus, dysregulation of this gene could impact synaptic protein function relevant to memory impairment [37,39]. Interestingly, we identified that ENST00000427926 (*CLTCL1*) may predict the number of perseverant errors in the WCST (Table 3), which assesses cognitive flexibility and executive function. *CLTCL1* regulates vesicular trafficking essential for synaptic communication. Hence, its dysregulation affects APP processing and contributes to Aβ accumulation observed in AD brains [29,38], which in turn impacts important cognitive processes.

*CBX7* is a chromatin modifier that regulates gene expression and may affect neuronal survival mechanisms [41,42]. Altered expression of mRNAs within this gene may disrupt these processes, leading to deficits in language and naming abilities, while associations with TMT performance could reflect involvement in executive function/processing speed [43,44,45]. Changes in mRNA expression may impair these cognitive domains, contributing to the observed deficits in TMT performance (Table 1).

Finally, the ENST00000331581 (*CADM1*) was found to predict MoCA (Table 3). Interestingly, this transcript was upregulated in individuals with AD and identified as a key predictor of AD diagnosis [8]. *CADM1* promotes synaptic adhesion and connectivity [29,46]. Thus, potential alterations in expression levels may impact synaptic integrity and memory function, both severely affected in AD pathology, and assessed by the MoCA test.

Previous studies have identified altered mRNA profiles in exosomes derived from AD patients compared to healthy controls [16,17,18,19], often focusing on blood and cerebrospinal fluid samples [47,48,49]. Our study builds upon this research by examining a cohort from Barranquilla, Colombia, with a unique genetic background and environmental exposure that differs from other AD communities in Colombia [50,51,52,53,54]. We found that specific mRNA transcripts were significantly correlated with performance on neuropsychological tests commonly used to assess cognitive function in AD, such as the BNT, TMT, and ROCFT (Figure 1 and Table 2). These correlations suggest potential mechanisms through which these transcripts may influence cognitive function in AD.

This study benefits from a well-characterized AD cohort and controls in Colombia, with comprehensive neuropsychological and advanced data analytics. Limitations include small sample size, potential regional bias, and a cross-sectional design. Future research should validate findings in larger, multi-centre, diverse cohorts using longitudinal designs to assess temporal relationships. Functional in vitro studies could clarify the causal role of identified mRNA transcripts in AD pathogenesis.

## 4. Materials and Methods

### 4.1. Participants

We recruited 30 participants (15 with a diagnosis of AD and 15 healthy controls) at the Instituto Colombiano de Neuropedagogía (ICN) in Barranquilla, Colombia, and collected data from clinical evaluations, family histories, comprehensive neurological and neuropsychological clinical examinations, and structured interviews. The ICN team determined the candidates’ eligibility based on the Montreal Cognitive Assessment (MoCA) test [55] and the inclusion criteria described elsewhere [7]. Individuals were classified as affected by AD if they had a Mini-Mental State Examination (MMSE) [56] between 0 and 18 points and met the DSM-5 criteria [57]. Individuals with other neurological or major psychiatric disorders, psychoactive substance use, excessive alcohol consumption, and inability to complete the clinical studies were excluded [7]. Healthy controls were non-family volunteers aged over 65, without suspected AD, and with an MMSE score between 19 and 29. Individuals with depression, mild cognitive impairment, dementia, other neurological disorders, major psychiatric illnesses, psychoactive substance use, or excessive alcohol consumption were excluded. The Universidad del Norte Ethics Committee approved this study (Project Approval Act #188 of 23 May 2019). Demographic and clinical data are summarized in Table 1.

### 4.2. Neuropsychological Assessment

We clinically characterized all participants using an exhaustive neuropsychological evaluation protocol described elsewhere [7,8]. In addition to the MoCA and MMSE tests, this protocol included the Boston Denomination Test [58,59], Rey–Osterrieth Complex Figure Test (ROCFT) [60], Rey Auditory Verbal Learning Test (RAVLT) [61], Trail Making Test (TMT) [62,63], Symbol Digit Modality Test (SDMT) [64], Stroop Color and Word Test [65], Token Test [66], Benton’s Visual Retention Test (BVRT) [67], Clock Drawing Test [68], Memory Scale subtest of the Wisconsin Card Testing Test (WCST) [69], Geriatric Depression Screening Test [70], Global Deterioration Scale (GDS) [71], Barthel Functional Index [72], and the Neuropsychiatric Inventory [73]. All participants’ age at the beginning of the study, sex, educational level, marital status, weight, and height were also recorded through the clinical history. In individuals with AD, the AD age of onset (ADAOO) was also defined following previous studies [74,75]. Missing data, a common feature of clinical studies, were handled using the imputation method implemented in the missForest [76,77] package for R [78]. Subsequent statistical analyses were performed on the imputed dataset.

### 4.3. RNA Isolation and Extraction

Blood samples were collected to isolate circulating exosomes following the protocol previously described [7]. Exosome isolation was performed using the Total Exosome Isolation Reagent (Thermo Fisher Scientific, San Francisco, CA, USA) according to the manufacturer’s instructions, with minor modifications standardized at the Universidad del Norte laboratories in Barranquilla, Colombia. Isolated exxosomes were characterized using scanning electron microscopy. RNA extraction from the exosomes was conducted using a laboratory-standardized acid phenol–chloroform method [7]. Extracted RNA was resuspended in 50 µL of RNase-free water and treated with DNase I (Thermo Fisher Scientific, San Francisco, CA, USA) according to the manufacturer’s protocol. RNA quality was assessed using a NanoDrop 2000 spectrophotometer (Thermo Fisher Scientific, San Francisco, CA, USA), measuring optical density (OD) ratios at 260/230 and 260/280 to ensure high-quality RNA suitable for downstream applications.

### 4.4. mRNA Microarray Study

A total of 30 RNA samples (15 from AD cases and 15 from healthy controls) were analyzed. RNA quality control, labelling, and hybridization followed Agilent’s singlx10-color microarray-based gene expression analysis protocol with minor modifications. Each RNA sample underwent reverse transcription to complementary DNA (cDNA), followed by amplification and transcription back to complementary RNA (cRNA). During this process, cyanine-3 (Cy3) fluorescent dye was incorporated using a random priming method. The labeled cRNAs were purified using the RNeasy Mini Kit (QIAGEN, Germantown, MD, USA) to eliminate reagent residues and excess dye. Quality control metrics included a cRNA concentration threshold of >1.65 μg and specific activity of >9 pmol Cy3/μg cRNA; samples failing these criteria were reprocessed.

For hybridization, 1 μg of labeled cRNA was fragmented, mixed with blocking and fragmentation buffers, and diluted with hybridization buffer. The hybridization solution was applied to lncRNA expression microarray plates and incubated for 17 h at 65 °C in an Agilent hybridization oven. Post-incubation, the arrays were washed and scanned using an Agilent G2505C scanner (Agilent Scientific Instruments, Santa Clara, CA, USA).

We used the Arraystar Human LncRNA Arrays V5 platform, which profiles 39,317 lncRNAs and 21,174 mRNA transcripts. Probes targeting specific exons or splice junctions ensured accurate transcript identification. Positive and negative control probes for housekeeping genes were included for quality assurance. Quantile normalization and data processing were performed using GeneSpring GX v12.1 software (Agilent Scientific Instruments, Santa Clara, CA, USA). Only mRNAs flagged as present or marginal in at least 15 of the 30 samples were selected for further analysis.

### 4.5. mRNA Signatures Linked to Neuropsychological Manifestations of AD

mRNAs correlated to neuropsychological manifestations of AD were identified using Generalized Linear Models (GLMs) [79]. For the *i*th neuropsychological variable *y_i_* (i = 1, 2, …, 25), a GLM of the form *y_i_* ~ mRNA*_j_* + AD + Age + Sex + Schooling was fitted to the data as implemented in R [78]. In this model, mRNA*_j_* corresponds to the expression of the *j*th mRNA (*j* = 1, 2, …, 16,585), AD is a binary variable indicating the diagnosis of the participant (0: control; 1: case), Age is the age of the individual at the beginning of the study and Schooling is the years of education. The family distribution, a main component of a GLM, was selected according to the nature of the neuropsychological variable. Thus, neuropsychological variables representing counts were modelled using a Poisson distribution, and those of continuous nature were modelled using a Gamma distribution to account for potential skewness. Subsequently, the estimated regression coefficient ${\hat{\beta }}_{j}$ associated with mRNA*_j_*, was extracted from the fitted model along with its standard error ${\hat{SE}}_{{\hat{\beta }}_{j}}$. Values of ${\hat{\beta }}_{j}>0$ implies that the expression of the *j*th mRNA is positively correlated with the neuropsychological variable; ${\hat{\beta }}_{j}<0$ implies that the expression of the *j*th mRNA is negatively correlated; and ${\hat{\beta }}_{j}=0$ implies that there is no correlation (*j* = 1, 2, …, 16,580). Under the null hypothesis, the *p*-value for the *j*th mRNA is calculated as $P_{j}=2Pr(t_{n-p}>|t_{j}|)$, where $t_{n-p}$ is a t distribution with *n* − *p* = 30 − 6 = 24 degrees of freedom and $t_{j}=\frac{{\hat{\beta }}_{j}}{{\hat{SE}}_{{\hat{\beta }}_{j}}}$ is the test statistic. The resulting *p*-values were corrected for multiple testing using Bonferroni’s method [80] and the false discovery rate (FDR) [81,82,83]. mRNAs corrected *p*-values < 5% were statistically significantly correlated with a particular neuropsychological variable.

### 4.6. Predictive Power of mRNAs in AD

The Predictive Power Score (PPS) evaluates the predictive relationships between variables, addressing limitations of traditional correlation by accommodating non-linear patterns, categorical data, and asymmetric associations [33]. Unlike correlation and GLM-based analyses, PPS identifies directional predictive strength. In addition, the PPS quantifies the performance of a Decision Tree model in predicting a target variable via out-of-sample validation, benchmarking against naive approaches. We used the PPS as implemented in the ppsr [34] package of R to quantify the prediction ability of mRNA*_j_* (*j* = 1, 2, …, 16,585) on the neuropsychological variable *y_i_* (*i =* 1, 2, 3, …,25).

## 5. Conclusions

Our study provides novel insights into the relationship between exosome-derived mRNA signatures and neuropsychological manifestations in AD. We have identified specific mRNA transcripts that correlate with cognitive performance. These findings advance our understanding of AD pathogenesis’ molecular mechanisms and open new avenues for developing non-invasive diagnostic tools and targeted therapies. Further research is needed to validate these findings and translate them into clinical applications, ultimately improving the diagnosis, treatment, and prevention of AD.

## Figures and Tables

**Table 1:** Neuropsychological characteristics of individuals included in this study.

Variable	Cases	Controls	W ^a^	*p*-Value
	**Mean (SD)**			
Age (years)	77.5 (8.5)	82.1 (8.6)	900	<0.001
MMSE	13.9 (9.5)	25.2 (5.6)	855	<0.001
MoCA	5.5 (5.3)	25.9 (2.9)	224	<0.001
FAST	4.5 (3.2)	2.5 (0.6)	19	<0.001
Boston Naming Test				
Spontaneous clues	14.1 (11.6)	37.5 (13.9)	200.5	<0.001
Semantic clues	0.7 (1.2)	1.3 (1.4)	138.5	0.248
Total score	14.8 (12.1)	38.7 (14.2)	201.5	<0.001
Verbal Fluency				
Letter “a”	3.4 (2.8)	11.2 (3.7)	212.5	<0.001
Letter “c”	4.5 (3.8)	8.7 (4)	177	0.008
Phonological fluency				
Letter ”a”	2.6 (3.4)	8.6 (4.8)	191	0.001
Letter “s”	2.8 (2.8)	8.3 (5.3)	179	0.006
Letter “f”	3.6 (3.8)	8.2 (5.8)	163.5	0.035
Trail Making Test				
Part A	115.5 (79.8)	109 (77)	101	0.648
Part B	145.4 (130.8)	233 (105)	157.5	0.063
Token test	14.1 (10)	26.2 (10.8)	187	0.002
Lawton and Brody test	1.7 (1.4)	0.3 (0.8)	175.5	0.003
ROCFT				
Copy	5.6 (9.2)	24.7 (13.5)	193	<0.001
Recall	1.3 (2.4)	6.3 (5.6)	181	0.004
AVMR, “Yes”	6.7 (6.4)	11.7 (4.1)	169.5	0.018
AVMR, “No”	7.3 (6.2)	11.9 (5.2)	163	0.033
Stroop test				
Words	33.2 (17.3)	60.1 (32.4)	178	0.007
Colours	20.3 (13)	39.4 (22.2)	170	0.018
Wisconsin Card Sorting Test				
Categories	0.7 (0.9)	2.6 (2.2)	170	0.015
NPE	25.8 (24.1)	20.5 (29.9)	89	0.339
Perseverant errors	26.1 (19.2)	18.9 (12.9)	87.5	0.309
Correct responses	25.1 (23.8)	42.8 (40.1)	137	0.319

^a^ Mann–Whitney–Wilcoxon non-parametric statistic. The reported *p*-value was not adjusted for covariates. AVMR: Auditory-verbal memory recognition; FAST: Functional Assessment Screening Tool; MMSE: Mini-Mental State Examination; MoCA: Montreal Cognitive Assessment; NPE: Non-perseverant errors; ROCFT: Rey–Osterrieth Complex Figure test.

**Table 2:** Top 10 mRNAs correlated with AD for each neuropsychological variable. Conventions as in Table 1.

Test	Transcript	Chr	Position *^a^*	Gene	$\hat{\beta }\;({\hat{SE}}_{\hat{\beta }})$	*p*	*p*_Bonferroni_
ROCFT							
Copy	ENST00000382830	21	31,962,424	*KRTAP22-2*	0.567 (0.076)	6.74 × 10^−14^	1.12 × 10^−9^
	ENST00000361033	20	2,796,948	*TMEM239*	−1.384 (0.185)	7.41 × 10^−14^	1.23 × 10^−9^
	ENST00000380210	9	21,349,834	*IFNA6*	0.396 (0.053)	8.14 × 10^−14^	1.35 × 10^−9^
	ENST00000216037	22	29,190,543	*XBP1*	0.475 (0.064)	1.06 × 10^−13^	1.76 × 10^−9^
	ENST00000398576	13	46,700,055	*LCP1*	−0.268 (0.037)	3.64 × 10^−13^	6.04 × 10^−9^
	ENST00000221566	19	2,754,712	*SGTA*	−0.992 (0.137)	4.74 × 10^−13^	7.86 × 10^−9^
	ENST00000334456	11	72,287,185	*PDE2A*	0.387 (0.054)	5.11 × 10^−13^	8.48 × 10^−9^
	ENST00000295201	2	95,537,188	*TEKT4*	1.205 (0.17)	1.29 × 10^−12^	2.14 × 10^−8^
	ENST00000360242	18	66,465,317	*CCDC102B*	−0.639 (0.091)	2.00 × 10^−12^	3.31 × 10^−8^
	ENST00000544413	12	121,416,552	*HNF1A*	−1.399 (0.2)	2.45 × 10^−12^	4.06 × 10^−8^
Recall	HBMT00000891055	20	47,127,407	*CATG00000053459.1*	−0.845 (0.169)	5.33 × 10^−7^	8.84 × 10^−3^
	ENST00000339480	1	35,225,342	*GJB4*	−0.852 (0.174)	1.03 × 10^−6^	1.70 × 10^−2^
	ENST00000545128	9	78,505,560	*PCSK5*	−0.972 (0.205)	2.08 × 10^−6^	3.45 × 10^−2^
	ENST00000398093	11	102,980,304	*DYNC2H1*	1.23 (0.261)	2.46 × 10^−6^	4.08 × 10^−2^
	ENST00000295201	2	95,537,188	*TEKT4*	1.375 (0.294)	2.95 × 10^−6^	4.89 × 10^−2^
	ENST00000378567	1	1,981,909	*PRKCZ*	−1.004 (0.215)	2.95 × 10^−6^	4.90 × 10^−2^
BNT							
Spontaneous Clues	ENST00000216487	14	92,980,118	*RIN3*	0.453 (0.081)	2.39 × 10^−8^	3.97 × 10^−4^
	ENCT00000457686	9	90,652,380	*CATG00000108922.1*	0.554 (0.101)	4.36 × 10^−8^	7.22 × 10^−4^
	ENCT00000061513	10	134,202,355	*CATG00000001242.1*	−0.44 (0.081)	5.67 × 10^−8^	9.40 × 10^−4^
	ENST00000219070	16	55,512,883	*MMP2*	−0.445 (0.084)	1.15 × 10^−7^	1.91 × 10^−3^
	ENCT00000228958	2	119,913,597	*CATG00000044356.1*	−0.788 (0.159)	6.86 × 10^−7^	1.14 × 10^−2^
	ENST00000234347	19	840,960	*PRTN3*	−0.443 (0.09)	8.98 × 10^−7^	1.49 × 10^−2^
	ENST00000210313	9	123,578,331	*PSMD5*	−0.243 (0.05)	9.45 × 10^−7^	1.57 × 10^−2^
	ENST00000216756	14	102,814,619	*CINP*	0.264 (0.054)	1.13 × 10^−6^	1.87 × 10^−2^
	ENST00000242819	13	52,436,117	*CCDC70*	−0.498 (0.103)	1.36 × 10^−6^	2.25 × 10^−2^
	ENST00000004531	8	17,396,286	*SLC7A2*	−0.366 (0.077)	2.07 × 10^−6^	3.43 × 10^−2^
Total	ENCT00000061513	10	134,202,355	*CATG00000001242.1*	−0.458 (0.08)	9.05 × 10^−9^	1.50 × 10^−4^
	ENCT00000457686	9	90,652,380	*CATG00000108922.1*	0.552 (0.099)	2.81 × 10^−8^	4.66 × 10^−4^
	ENST00000004531	8	17,396,286	*SLC7A2*	−0.396 (0.076)	1.57 × 10^−7^	2.60 × 10^−3^
	ENCT00000228958	2	119,913,597	*CATG00000044356.1*	−0.808 (0.155)	1.89 × 10^−7^	3.14 × 10^−3^
	ENCT00000380453	6	168,062,372	*CATG00000086946.1*	0.377 (0.075)	4.66 × 10^−7^	7.73 × 10^−3^
	ENCT00000200728	19	3,630,183	*CATG00000038258.1*	0.264 (0.055)	1.35 × 10^−6^	2.25 × 10^−2^
	ENCT00000029805	1	109,072,893	*CATG00000070137.1*	0.256 (0.054)	1.78 × 10^−6^	2.96 × 10^−2^
	ENCT00000447643	9	88,474,187	*CATG00000105979.1*	−0.342 (0.073)	2.41 × 10^−6^	4.00 × 10^−2^
	ENCT00000424376	8	41,121,640	*CATG00000098647.1*	0.351 (0.075)	2.47 × 10^−6^	4.10 × 10^−2^
	ENCT00000370852	6	29,601,041	*CATG00000083443.1*	0.261 (0.056)	2.78 × 10^−6^	4.61 × 10^−2^
*TMT*							
Part A	ENST00000228506	12	121,124,672	*MLEC*	−43.181 (5.064)	1.00 × 10^−8^	1.66 × 10^−4^
Part B	MICT00000221720	20	60,942,556	*CATG00000053936.1*	−86.275 (11.342)	7.61 × 10^−8^	1.26 × 10^−3^
	ENST00000263246	22	43,265,777	*PACSIN2*	−69.407 (11.271)	2.31 × 10^−6^	3.83 × 10^−2^
Token test	MICT00000383608	Y	18,943,870	*CATG00000114908.1*	−0.71 (0.134)	1.15 × 10^−7^	1.91 × 10^−3^
	ENST00000296043	4	77,356,253	*SHROOM3*	−0.663 (0.13)	3.16 × 10^−7^	5.24 × 10^−3^
	ENST00000380534	9	18,927,656	*SAXO1*	0.728 (0.142)	3.16 × 10^−7^	5.24 × 10^−3^
Stroop test							
Colours	ENCT00000309252	3	134,030,483	*CATG00000066161.1*	−0.565 (0.098)	7.53 × 10^−9^	1.25 × 10^−4^
	ENST00000216037	22	29,190,543	*XBP1*	0.251 (0.044)	1.06 × 10^−8^	1.76 × 10^−4^
	ENST00000174618	17	2,287,354	*MNT*	0.225 (0.04)	1.44 × 10^−8^	2.39 × 10^−4^
	ENST00000215743	22	24,115,006	*MMP11*	0.439 (0.085)	2.61 × 10^−7^	4.34 × 10^−3^
	ENST00000221566	19	2,754,712	*SGTA*	−0.456 (0.09)	4.03 × 10^−7^	6.68 × 10^−3^
	ENST00000223369	7	44,240,648	*YKT6*	−0.349 (0.07)	6.00 × 10^−7^	9.95 × 10^−3^
	ENST00000216133	22	39,526,777	*CBX7*	0.293 (0.062)	2.17 × 10^−6^	3.60 × 10^−2^
	ENST00000231228	5	158,741,791	*IL12B*	−0.22 (0.047)	2.32 × 10^−6^	3.85 × 10^−2^
	ENCT00000193672	18	60,987,564	*CATG00000036339.1*	−0.328 (0.07)	2.59 × 10^−6^	4.29 × 10^−2^
	ENCT00000431277	8	144,959,539	*CATG00000101329.1*	−0.381 (0.082)	2.98 × 10^−6^	4.94 × 10^−2^
Words	ENST00000171111	19	10,596,796	*KEAP1*	0.271 (0.043)	2.40 × 10^−10^	3.98 × 10^−6^
	ENST00000201647	19	55,587,269	*EPS8L1*	−0.369 (0.065)	1.46 × 10^−8^	2.43 × 10^−4^
	ENST00000250160	8	134,203,282	*WISP1*	−0.252 (0.047)	7.78 × 10^−8^	1.29 × 10^−3^
	ENST00000251453	19	39,923,847	*RPS16*	0.334 (0.066)	4.46 × 10^−7^	7.39 × 10^−3^
	ENST00000225698	17	5,336,097	*C1QBP*	−0.223 (0.045)	6.24 × 10^−7^	1.03 × 10^−2^
	ENCT00000309252	3	134,030,483	*CATG00000066161.1*	−0.371 (0.077)	1.28 × 10^−6^	2.12 × 10^−2^
	ENST00000230588	6	46,761,127	*MEP1A*	−0.238 (0.049)	1.43 × 10^−6^	2.37 × 10^−2^
	ENST00000225567	17	45,000,486	*GOSR2*	−0.345 (0.072)	1.82 × 10^−6^	3.03 × 10^−2^
	ENST00000216254	22	41,865,129	*ACO2*	0.267 (0.056)	1.96 × 10^−6^	3.25 × 10^−2^
WCST							
Correct responses	ENCT00000012768	1	156,638,559	*CATG00000020670.1*	0.736 (0.085)	4.37 × 10^−18^	7.25 × 10^−14^
	ENCT00000000389	1	1,874,595	*CATG00000071025.1*	−0.679 (0.08)	2.46 × 10-17	4.09 × 10^−13^
	ENCT00000004417	1	38,891,158	*CATG00000115972.1*	0.19 (0.023)	4.03 × 10^−16^	6.68 × 10^−12^
	ENCT00000000232	1	1,138,890	*CATG00000019495.1*	−0.566 (0.082)	4.07 × 10^−12^	6.75 × 10^−8^
	ENCT00000000644	1	4,077,807	*CATG00000116876.1*	−0.654 (0.095)	6.99 × 10^−12^	1.16 × 10^−7^
	ENCT00000002816	1	25,046,862	*CATG00000062929.1*	−0.389 (0.061)	1.34 × 10^−10^	2.22 × 10^−6^
	ENCT00000001323	1	10,960,567	*CATG00000015125.1*	0.479 (0.078)	6.95 × 10^−10^	1.15 × 10^−5^
	ENCT00000003570	1	30,996,263	*CATG00000087839.1*	0.31 (0.051)	1.32 × 10^−9^	2.19 × 10^−5^
	ENCT00000002257	1	19,234,224	*CATG00000038794.1*	0.513 (0.092)	2.23 × 10^−8^	3.70 × 10^−4^
	ENCT00000004031	1	35,331,806	*CATG00000107162.1*	−0.287 (0.059)	1.02 × 10^−6^	1.69 × 10^−2^
NPE	ENCT00000000276	1	1,284,939	*CATG00000033020.1*	−1.178 (0.137)	1.08 × 10^−17^	1.80 × 10^−13^
	ENCT00000020781	1	1,964,944	*CATG00000043697.1*	−0.899 (0.109)	1.47 × 10^−16^	2.43 × 10^−12^
	ENCT00000005948	1	53,558,713	*CATG00000001175.1*	0.614 (0.083)	1.37 × 10^−13^	2.28 × 10^−9^
	ENCT00000020405	1	984,575	*CATG00000042982.1*	−0.479 (0.068)	2.20 × 10^−12^	3.64 × 10^−8^
	ENCT00000004031	1	35,331,806	*CATG00000107162.1*	−0.426 (0.069)	6.99 × 10^−10^	1.16 × 10^−5^
	ENCT00000000644	1	4,077,807	*CATG00000116876.1*	−0.55 (0.102)	7.09 × 10^−8^	1.18 × 10^−3^
	ENCT00000020445	1	1,087,776	*CATG00000043113.1*	0.752 (0.14)	7.36 × 10^−8^	1.22 × 10^−3^
	ENCT00000002816	1	25,046,862	*CATG00000062929.1*	−0.374 (0.07)	9.87 × 10^−8^	1.64 × 10^−3^
	ENCT00000018210	1	225,841,146	*CATG00000037190.1*	0.258 (0.051)	3.41 × 10^−7^	5.65 × 10^−3^
	ENCT00000029656	1	104,998,991	*CATG00000069026.1*	−0.543 (0.115)	2.43 × 10^−6^	4.03 × 10^−2^
Perseverant errors	ENCT00000228958	2	119,913,597	*CATG00000044356.1*	−0.731 (0.135)	6.09 × 10^−8^	1.01 × 10^−3^
	ENCT00000045141	10	38,027,225	*CATG00000112585.1*	0.453 (0.084)	7.49 × 10^−8^	1.24 × 10^−3^
	ENCT00000272151	21	46,270,031	*CATG00000056264.1*	−0.37 (0.071)	1.83 × 10^−7^	3.03 × 10^−3^
	ENCT00000263490	20	61,077,116	*CATG00000053945.1*	0.626 (0.124)	4.77 × 10^−7^	7.91 × 10^−3^
	ENCT00000474207	X	2,742,248	*CATG00000112964.1*	−0.361 (0.073)	6.42 × 10^−7^	1.07 × 10^−2^
	ENCT00000431277	8	144,959,539	*CATG00000101329.1*	0.422 (0.088)	1.47 × 10^−6^	2.44 × 10^−2^
	ENCT00000113077	13	55,351,449	*CATG00000014934.1*	0.49 (0.103)	1.92 × 10^−6^	3.18 × 10^−2^
	ENST00000055682	X	73,952,691	*NEXMIF*	−0.323 (0.068)	2.28 × 10^−6^	3.78 × 10^−2^
	ENST00000013807	19	45,916,692	*ERCC1*	−0.383 (0.081)	2.30 × 10^−6^	3.81 × 10^−2^
	ENCT00000202697	19	17,008,342	*CATG00000038771.1*	0.393 (0.083)	2.43 × 10^−6^	4.02 × 10^−2^

*^a^* UCSC GRCh37/hg19 coordinates. BNT: Boston Naming Test.

**Table 3:** mRNAs with the highest PPS for each neuropsychological test. Conventions as in Table 2.

Variable	Transcript	Chr	Position	Gene	PPS
AVMR					
No	ENST00000295268	4	98,480,027	*STPG2*	0.295
	ENST00000474844	1	46,805,849	*NSUN4*	0.295
	ENST00000274773	5	180,620,924	*TRIM7*	0.293
	ENST00000623276	6	28,234,931	*ZSCAN26*	0.289
	ENST00000317907	2	32,853,129	*TTC27*	0.273
Yes	ENST00000307395	3	128,779,610	*GP9*	0.347
	ENST00000299608	18	66,340,925	*TMX3*	0.331
	ENST00000609883	X	71,347,574	*RTL5*	0.329
	ENST00000343053	9	140,149,625	*NELFB*	0.322
	ENST00000409299	20	32,290,560	*PXMP4*	0.316
BNT					
Spontaneous clues	ENST00000274773	5	180,620,924	*TRIM7*	0.391
	ENST00000361900	15	75,287,939	*SCAMP5*	0.298
	ENST00000375581	13	113,760,121	*F7*	0.287
	ENST00000368751	1	153,065,611	*SPRR2E*	0.274
	ENST00000524140	19	16,830,791	*NWD1*	0.264
Semantic clues	ENST00000517870	1	53,099,016	*SHISAL2A*	0.374
	ENST00000622339	1	104,159,433	*AMY2A*	0.361
	ENST00000330233	14	105,952,654	*CRIP1*	0.336
	ENST00000254691	5	40,841,286	*CARD6*	0.320
	ENST00000409790	16	11,038,345	*CLEC16A*	0.311
Total	ENST00000274773	5	180,620,924	*TRIM7*	0.386
	ENST00000361900	15	75,287,939	*SCAMP5*	0.304
	ENST00000375581	13	113,760,121	*F7*	0.292
	ENST00000262426	16	86,544,133	*FOXF1*	0.275
	ENST00000323853	2	96,940,074	*SNRNP200*	0.267
FAST	ENST00000378165	10	15,149,865	*NMT2*	0.271
	ENST00000311550	15	26,788,693	*GABRB3*	0.227
	ENST00000611257	17	34,493,061	*TBC1D3B*	0.209
	ENST00000643399	10	71,038,252	*HK1*	0.167
	ENST00000290158	17	45,727,204	*KPNB1*	0.160
Lawton and Brody	ENST00000216442	14	67,804,788	*ATP6V1D*	0.306
	ENST00000297770	8	68,334,307	*CPA6*	0.308
	ENST00000318225	3	126,268,516	*C3orf22*	0.315
	ENST00000250056	17	6,347,761	*PIMREG*	0.341
	ENST00000299367	6	31,895,254	*C2*	0.430
MMSE	ENST00000528494	11	46,639,150	*ATG13*	0.221
	ENST00000304385	4	153,539,784	*TMEM154*	0.232
	ENST00000394152	7	99,214,571	*ZSCAN25*	0.240
	ENST00000262426	16	86,544,133	*FOXF1*	0.247
	ENST00000274773	5	180,620,924	*TRIM7*	0.292
MoCA	ENST00000311550	15	26,788,693	*GABRB3*	0.647
	ENST00000343289	10	104,847,775	*NT5C2*	0.439
	ENST00000340116	18	6739	*ENOSF1*	0.428
	ENST00000331581	11	115,047,015	*CADM1*	0.425
	FTMT26400003890	16	67,267,859	*FHOD1*	0.423
Phonological fluency					
Letter “a”	ENST00000355790	10	72,058,729	*LRRC20*	0.255
	ENST00000611257	17	34,493,061	*TBC1D3B*	0.235
	ENST00000382258	13	24,153,499	*TNFRSF19*	0.224
	ENST00000379731	9	33,110,635	*B4GALT1*	0.224
	ENST00000374510	9	113,065,867	*TXNDC8*	0.222
Letter “f”	ENST00000355790	10	72,058,729	*LRRC20*	0.297
	ENST00000296043	4	77,356,253	*SHROOM3*	0.277
	ENST00000259883	6	28,249,349	*PGBD1*	0.242
	ENST00000340913	12	54,674,539	*HNRNPA1*	0.231
	HBMT00001348771	7	140,772,165	*TMEM178B*	0.228
Letter “s”	ENST00000284268	5	14,704,909	*ANKH*	0.224
	ENST00000598357	19	45,842,445	*L47234.1*	0.215
	ENST00000222990	7	2,291,405	*SNX8*	0.211
	ENST00000355790	10	72,058,729	*LRRC20*	0.206
	ENST00000305366	3	149,086,809	*TM4SF1*	0.206
ROCFT					
Copy	ENCT00000073979	11	1,403,334	*BRSK2*	0.336
	ENST00000274773	5	180,620,924	*TRIM7*	0.327
	ENST00000310248	12	48,595,866	*OR10AD1*	0.300
	ENST00000418703	12	110,220,890	*TRPV4*	0.298
	ENST00000300433	17	48,348,767	*TMEM92*	0.293
Recall	ENST00000334571	14	74,416,996	*COQ6*	0.330
	ENST00000578812	17	8,282,463	*RPL26*	0.316
	ENST00000310248	12	48,595,866	*OR10AD1*	0.301
	ENST00000358607	19	18,699,535	*REX1BD*	0.288
	ENST00000382723	4	4,861,393	*MSX1*	0.285
Stroop test					
Colors	ENST00000278483	11	86,013,265	*HIKESHI*	0.323
	ENST00000335852	1	156,213,112	*PAQR6*	0.264
	ENST00000283928	7	27,870,192	*JAZF1*	0.237
	MICT00000155430	17	76,171,134	*TK1*	0.230
	ENST00000300093	16	23,690,143	*PLK1*	0.215
Words	MICT00000155430	17	76,171,134	*TK1*	0.249
	ENST00000278483	11	86,013,265	*HIKESHI*	0.217
	ENST00000540200	17	26,674,203	*POLDIP2*	0.205
	ENST00000378981	X	30,261,847	*MAGEB1*	0.204
	HBMT00000611233	17	75,249,896	*CATG00000032482.1*	0.194
TMT					
Part A	ENST00000302823	2	204,732,509	*CTLA4*	0.250
	ENST00000428112	1	47,024,371	*MKNK1*	0.238
	MICT00000156619	17	79,759,048	*GCGR*	0.219
	ENST00000291700	21	48,018,875	*S100B*	0.216
	ENST00000354905	3	190,146,444	*TMEM207*	0.215
Part B	ENST00000304385	4	153,539,784	*TMEM154*	0.421
	ENST00000274773	5	180,620,924	*TRIM7*	0.414
	ENST00000241051	11	33,037,410	*DEPDC7*	0.302
	ENST00000498273	1	62,660,503	*L1TD1*	0.283
	ENST00000398399	3	86,987,119	*VGLL3*	0.273
Token test	ENST00000274773	5	180,620,924	*TRIM7*	0.254
	ENST00000304385	4	153,539,784	*TMEM154*	0.215
	ENST00000278483	11	86,013,265	*HIKESHI*	0.212
	ENST00000375581	13	113,760,121	*F7*	0.208
	ENST00000301838	11	70,049,269	*FADD*	0.202
Verbal Fluency					
Letter “a”	ENST00000375581	13	113,760,121	*F7*	0.298
	ENST00000379052	6	17,281,577	*RBM24*	0.272
	ENST00000397095	7	1,094,921	*GPR146*	0.271
	ENST00000311550	15	26,788,693	*GABRB3*	0.262
	ENST00000427500	1	155,204,350	*GBA*	0.262
Letter “c”	ENST00000274773	5	180,620,924	*TRIM7*	0.286
	ENST00000375259	9	99,082,992	*SLC35D2*	0.226
	ENST00000367175	1	204,586,298	*LRRN2*	0.220
	ENST00000611870	16	76,311,176	*CNTNAP4*	0.215
	ENST00000457091	7	6,537,405	*GRID2IP*	0.205
WCST					
Categories	ENST00000256495	3	5,020,801	*BHLHE40*	0.316
	HBMT00000611233	17	75,249,896	*CATG00000032482.1*	0.285
	ENST00000379731	9	33,110,635	*B4GALT1*	0.264
	ENST00000230640	5	54,603,588	*MTREX*	0.254
	ENST00000404371	2	10,923,519	*PDIA6*	0.245
Correct responses	ENST00000230640	5	54,603,588	*MTREX*	0.291
	ENST00000281961	2	39,893,059	*TMEM178A*	0.281
	ENST00000243253	3	127,771,212	*SEC61A1*	0.268
	ENST00000453960	X	153,295,685	*MECP2*	0.267
	ENST00000608842	22	18,893,866	*DGCR6*	0.266
NPE	ENST00000260723	10	124,030,821	*BTBD16*	0.252
	ENST00000360428	18	28,569,974	*DSC3*	0.249
	ENST00000267436	14	50,709,152	*L2HGDH*	0.245
	ENST00000345080	6	105,404,923	*LIN28B*	0.241
	ENST00000292907	19	36,641,824	*COX7A1*	0.237
Perseverant errors	ENST00000255465	13	37,006,495	*CCNA1*	0.291
	ENST00000541135	11	61,197,528	*AP003108.2*	0.239
	ENST00000375460	1	17,575,593	*PADI3*	0.238
	ENST00000305632	7	72,981,863	*TBL2*	0.234
	ENST00000427926	22	19,166,986	*CLTCL1*	0.222

## Data Availability

The data presented in this study are available upon reasonable request from the corresponding authors. They are not publicly available due to the ongoing nature of the study and our commitment to protecting the privacy and confidentiality of our patients.

## Supplementary Materials

The following supporting information can be downloaded at: https://www.mdpi.com/article/10.3390/ijms26104897/s1.

## Abbreviations

The following abbreviations are used in this manuscript:

AD	Alzheimer’s Disease
ADAOO	Alzheimer’s Disease Age of Onset
AVMR	Auditory–Verbal Memory Recognition
Aβ	Amyloid-beta
BNT	Boston Naming Test
circRNA	Circular RNA
EVs	Extracellular Vesicles
FAST	Functional Assessment Screening Tool
GLM	Generalized Linear Model
lncRNA	Long Non-Coding RNA
MMSE	Mini-Mental State Examination
MoCA	Montreal Cognitive Assessment
mRNA	Messenger RNA
PPS	Predictive Power Score
ROCFT	Rey–Osterrieth Complex Figure Test
TMT	Trail Making Test
WCST	Wisconsin Card Sorting Test
